# Can Assessment of Rheological Properties of Whole Blood and Plasma Be Useful in the Diagnosis of Tinnitus? A Pilot Study

**DOI:** 10.3390/ijerph20031977

**Published:** 2023-01-20

**Authors:** Anna Marcinkowska-Gapińska, Barbara Maciejewska, Anna Majewska, Weronika Kawałkiewicz, Marta Urbaniak-Olejnik, Wawrzyniec Loba, Olgierd Stieler, Dariusz Komar, Leszek Kubisz, Michał Karlik, Dorota Hojan-Jezierska

**Affiliations:** 1 Department of Biophysics, Poznan University of Medical Sciences, 61-701 Poznań, Poland; 2 Department and Clinic of Phoniatrics and Audiology, Poznan University of Medical Sciences, 61-701 Poznań, Poland; 3 Department of Hearing Healthcare Profession, Poznan University of Medical Sciences, 61-701 Poznań, Poland

**Keywords:** tinnitus, hemorheology, blood viscosity, plasma viscosity

## Abstract

Tinnitus is a sensation of ringing in the ears in the absence of any physical source in the environment. Between 9–35% of adults experience some form of tinnitus. Common causes of tinnitus include noise, head injury, ototoxic substances, as well as disorders of blood and blood vessels. Vascular causes include: head—neck tumours, turbulent blood flow, problems with blood supply and inner ear cell damage. The aspect of rheology in terms of tinnitus has not been described yet. In the present study, which comprised 12 patients aged 30 to 74 years presenting with tinnitus, rheological properties of whole blood and plasma were assessed. All the subjects underwent audiological and neurological evaluation. The Quemada model was used to describe the variability of red blood cell shape, as well as their tendency to form aggregates. On the basis of the experimental study, statistically different results of haemorheological measurements were observed in the evaluated group in comparison to a reference group.

## 1. Introduction

Tinnitus is a sound sensation perceived by the patient in the absence of an acoustic stimulus in the environment. The condition is a challenge in the medical practice due to the heterogeneous etiology and the chronic nature of the complaint, as well as difficulties in effective treatment which is often short-lived and has delayed effects. Although tinnitus itself deteriorates the quality of life, typically it represents just the first symptom, or one of the main symptoms of various diseases [1]. Tinnitus often accompanies a variety of other hearing disorders but sometimes it occurs in individuals with normal hearing [2]. Such persistent, annoying noises impair speech understanding and make it difficult to concentrate and perform daily activities, as well as disturb night-time rest. In the most severe cases, tinnitus may also be accompanied by increased depression and anxiety [3]. Although the prevalence of tinnitus is high, no standards or recommendations for management have been developed up to date [4]. The recent studies provide evidence that cognitive behavioural therapy has a positive effect on tinnitus [5]. It is estimated that 9–35% of adults experience tinnitus temporarily, or permanently [6]. In fact, 30–35% of the world population complains of tinnitus (Poland 20%, Sweden 14.2%, England 9.7%), with 80% of cases resulting from cochlear damage [7,8]. It is experienced in one ear, both ears or inside the head, and is heard intermittently, or continuously. Furthermore, tinnitus may have a constant or variable level of masking, and its intensity, pitch and character may change over the course of their duration [9]. In terms of duration, tinnitus is classified into acute (up to 3 months), subacute (4–12 months) or chronic, which lasts more than a year [10,11]. It is also divided into objective and subjective tinnitus. When tinnitus is audible to the third parties, it is referred to as objective. It occurs rarely and does not appear in this study. Subjective tinnitus refers to the sensation of sound, a phantom phenomenon, resulting from the perception of an altered signal coming from the damaged auditory pathways to the auditory cortex, i.e., an erroneous generation of information in the auditory organ. The mechanism of its generation and perception is not fully understood, and may be caused by various factors, such as: noise, ototoxic substances, head and neck area trauma, but also by cardiovascular diseases inducing flow disorders, or by microcirculation disorders, e.g., in the course of metabolic diseases [12].

The analysis of blood flow as well as the phenomena accompanying this flow is es-sential in the course of various diseases, such as cerebrovascular disorders, coronary in-sufficiency, myocardial infarction, congestion, diabetes mellitus, Raynaud’s disease, is-chaemic limb disease, anaemia, or neoplastic diseases. In fact, circulatory disorders may be caused by factors related to peripheral blood supply disorders as well as to disturbed physicochemical properties of blood [13,14,15,16]. Hemorheology is a science investigating blood flow in blood vessels and phenomena associated with this flow. Moreover, haema-tocrit value, whole blood viscosity, plasma viscosity and erythrocyte deformability and aggregability are the determinants of blood flow [13,14,15,16]. These factors, in addition to shear rate and vessel diameter, constitute the main parameters affecting whole blood viscosity and thus blood flow in blood vessels [13]. According to the literature, plasma viscosity is affected by lipids in addition to protein fraction, such as fibrinogen and immunoglobulins [17,18]. On the other hand, Irace et al. demonstrated that LDL cholesterol and HDL cholesterol affect plasma viscosity, but not blood viscosity [19]. They found that triglyceride concentrations up to 400 mg/dl had no significant effect, at least in apparently healthy subjects, at the shear rates used in their study. Furthermore, they also noted that the effect of LDL and HDL cholesterol on plasma viscosity appears to be quite limited [19]. On the other hand, Nara et al. [20] and Dujovne et al. [21] observed that the lipid composition of the erythrocyte membrane affects its stiffness, and an increase in cholesterol concentration in the red blood cell membrane, in vitro, also leads to its stiffness. In fact, Watts et al. [22] found in their study an association between increased erythrocyte stiffness and altered platelet adhesiveness.

In patients with hearing impairment, the influence of blood rheological parameters on the risk of developing sudden hearing loss or hearing disorders has been observed [23,24], as well as the association of tinnitus with other cardiovascular diseases [25,26]. Studies aiming to identify the markers of tinnitus occurrence and prognosis have shown an association between hearing impairment and dyslipidaemia or comorbidities, such as diabetes [27,28,29].

The aim of the present study was to analyse the values of hemorheological parame-ters and the selected biochemical parameters in patients reporting chronic, subjective tin-nitus. No such studies have been found in the literature and the recent results of haemorheological differences in patients with neurological diseases [30] seems to justify this idea.

## 2. Materials and Methods

The study and analysis of the hemorheological parameters comprised a total of 12 patients, including 10 women and 2 men, aged between 30 and 74 years, suffering from chronic tinnitus (mean age 52 ± 5). In the study group, audiological (pure tone audiometry, impedance audiometry, otoacoustic emissions) and neurological assessments were performed, which varied individually depending on the co-existing diseases.

Only the subjects with sensorineural hearing loss diagnosed on the basis of the audiological tests were qualified for the study. Conductive hearing loss (i.e., the presence of cochlear reserve in pure tone audiometry and a tympanometric curve other than type Ain impedance audiometry) constituted an exclusion criterion.

The general clinical aspects of tinnitus were assessed using the THI tinnitus handicap index questionnaire. Tinnitus intensity was expressed using the VAS scale (0–10). To establish the relative intensity of tinnitus (i.e., loud, soft), we used a visual analogue scale like that used for measuring pain intensity. Tinnitus characterization was performed by the comparative method—the only one available and possible for subjective tinnitus. We asked patients to indicate the sound generated by a synthesizer that was as close as possible in terms of loudness and pitch to their tinnitus. The duration of the tinnitus was determined by the patient and was difficult to specify—the most common answer was ‘several years’.

A single collection of blood for hemorheological testing was performed on patients with the use of up to 5 mL of EDTA anticoagulant (1.6 mg EDTA/mL blood). The viscosity of whole blood and plasma was determined using a Contraves LS40 oscillatory-rotational rheometer at 37 °C, with decreasing shear rate γ′ in the range 100–0.01 s−1 over 5 min. Plasma viscosity was determined by linear regression from the shear stress/shear rate relationship. For each blood sample tested, the haematocrit value was determined using the standard method. The erythrocyte susceptibility to aggregation and deformation was analysed indirectly using Quemada’s mathematical rheological model (equations 1 and 2) by comparing numerical values of the model parameters: k_0_, k_∞_ and γ′_c_ [31]. The variables k_∞_ and k_0_ in the Quemada formula (1) are interpreted as measures of the erythrocyte stiffness and the degree of aggregation, whereas k_Q_ corresponds to the so-called erythrocyte intrinsic viscosity and, through the relation k_Q_(γ′) in the Quemada rheological formula (2), it accounts for erythrocyte shape variation. The variables τ(γ′), η_p_ and Hct denote shear stress as a function of shear rate, plasma viscosity and haematocrit, respectively [16,31,32].

(1) $$k_{Q}=\frac{k_{0}+k_{\infty }\left(\gamma \prime /{\gamma \prime }_{c}\right)1/2}{1+{\left(\gamma \prime /\gamma \prime_{c}\right)}^{1/2}}$$

(2) $$\tau \left(\gamma \prime \right)=\eta_{p}{\left[1-\frac{1}{2}k_{Q}\cdot Hct\right]}^{-2}\cdot \gamma \prime$$

The sample group is small. One of the reasons for this was the unwillingness of the patients to consent to the examination. The anxious personality trait of the patient with tinnitus, which is a consequence of the chronicity of the ailment in the absence of a definitive diagnosis, makes cooperation with them difficult because of reluctance towards unknown and non-standard tests.

## 3. Results

Table 1 presents the results of measurements including whole-blood viscosity values (η) for four selected shear rates (γ′) of the tested blood samples from the selected group of patients, as well as the results of plasma viscosity measurements (η_p_) and the mean value of haematocrit calculated on the basis of the value determined for each collected blood sample. The mean values of the Quemada model rheological parameters: k_0_, k_∞_ and γ′_c_ calculated on the basis of rotational measurements of the tested blood samples are presented in Table 1. The mean value of haematocrit was 0.44 ± 0.01%. The standard deviation of the mean value (SE) was presented as the measurement uncertainty for the data in Table 1. The mean values of the determined biochemical parameters in the studied group of patients are shown in Table 2.

The A-type tympanometric curve was recorded in all patients. No spontaneous otoacoustic emission suggesting an audiological origin of the noise was recorded in any case. Nevertheless, auditory thresholds varied. Table 3 presents the characteristics of selected audiological parameters and a list of comorbidities for each patient in the study group.

At the standard statistical significance level (0.05), no correlation was found between whole blood viscosity and either plasma viscosity, the Quemada model parameters, such as γ′_c_ and k_0_, or fibrinogen levels in the study group. Additionally, there were no correlations at the standard statistical significance level (0.05) between whole-blood viscosity and plasma viscosity, as well as individual biochemical parameters.

A reduced value of whole-blood viscosity in the range of low shear rates (γ′ = 0.1 and 1 s^−1^) was observed when comparing the obtained values of the hemorheological parameters with those obtained using the same measurement method published as control groups for neurological patients [30,33]. Individual analysis of the results indicates an increased tendency of erythrocytes to aggregate, although no statistically significant difference was found due to the large dispersion of results and the small sample size.

## 4. Discussion

The sample size was small. However, it should be noted that this is a preliminary study. It was designed to observe any hemorheological changes in a group of patients with tinnitus. The long-range goal was to test whether rheological measurements could find their justification for use in the eventual diagnostic process. The scattering of results indicates the need to enlarge the group and standardize it regarding the age, additional diseases, and the nature of hearing loss.

The large dispersion in the mean values of hemorheological parameters may be due to the relatively large variation due to comorbidities, where differences in hemorheological parameter values were also observed [34].

It is worth noting that a number of different diseases can also contribute to the occurrence of tinnitus. In fact, tinnitus can be caused by local diseases of the inner ear, such as presbyacusis or senile deafness, noise exposure, and Meniere’s disease. Similarly, temporomandibular joint disorders, due to the proximity of the ear and changes in the position of the head of the mandibular condyle process, may adversely affect the neurovascular bundle in the mandibular region [35]. However, they may also be associated with non-otological diseases affecting not only the hearing organ, but also the vascular system, and thus may modify the rheological properties of blood. Certain medications adversely affect the inner ear, including aminoglycoside antibiotics and drugs used in chemotherapy. The study group included users of non-steroidal anti-inflammatory drugs (NSAIDs), acetylsalicylic acid—for back pain and headache—and diuretics for hypertension and heart failure.

It has been found that adequate microcirculation is essential for optimal inner ear function. Therefore, ischaemia and, consequently, hypoxia significantly affect cochlear function [36,37,38]. Moreover, anaemia, hypertension and hypotension, as well as atherosclerotic lesions in the vessels, may lead to the development of tinnitus. All of the abovementioned factors alter the quality of blood flow in the blood vessels. Additionally, it is generally accepted that blood viscosity depends on several factors, including: the number of morphotic elements, particularly erythrocytes (haematocrit); the concentration of haemoglobin in erythrocytes; the content and concentration of haemoglobin in the blood cell, and its volume and shape; as well as on the level of proteins in the plasma. As a result, anaemia or hypoproteinaemia are characterised by a reduced blood viscosity. In fact, increased fibrinogen levels and blood and plasma viscosity accompany many risk factors for atherosclerosis, such as diabetes, hypertension, obesity, male gender, age, smoking and renal failure. The results of the study reflecting these observations indicate that the aforementioned risk factors can be narrowed down to their impact on the rheological properties of blood. In the study group, more than half of the patients presented at least one of the abnormalities mentioned above (Table 3). Nevertheless, it is interesting to note the cases with no hearing impairment and no vascular disease risk factors in the history, which were diagnosed as psychogenic tinnitus (5 patients). According to the literature, chronic stress activating the sympathetic nervous system leads to a reduced flow in the microcirculation as a result of vasospasm, increased blood viscosity, increased concentration of proteins, fibrinogen interleukin 1, increased leukocyte level, and increased platelet aggregation. As a result, vasoconstriction, haemoconcentration, and a reduced vascular flow are observed [39].

The haematocrit value was determined for each blood sample collected from a patient complaining of tinnitus. Haematocrit is one of the factors affecting haemorrhagic properties, and therefore it is essential that the study eliminates this factor as a potential cause of the observed differences between the study groups [31]. The mean result obtained in the study group of patients was within the physiologically normal range and did not account for the reduced viscosity values in the low shear rate range in the study group (Table 1), as compared to the groups of patients not complaining of tinnitus [30,33].

The measurement of whole-blood viscosity, plasma viscosity and the determination of the tendency of erythrocytes to aggregate and deform is aimed at determining the conditions of blood flow and accounting for the phenomena which are associated with this flow. One of the crucial phenomena is the formation and breakdown of erythrocyte aggregates, as well as their ability to deform and orient. In the low shear rate range, erythrocyte aggregation is the dominant process, whereas in the high shear rate range, erythrocyte deformation is dominant [13,14,15]. The results obtained in this study (i.e., lower whole-blood viscosity value in the range of low shear velocity) may indicate aggregation disorders in patients suffering from tinnitus. The aim of research regarding the analysis of phenomena occurring during blood flow is to understand the mechanisms regulating these processes in living organisms and, consequently, to understand the causes of vascular diseases. Numerous data indicate the importance of hemorheological properties in the course of vascular diseases, which usually result from atherosclerotic disorders (hypertension, ischaemic heart disease, narrowing of the cephalic arteries, ischaemic lesions in the central nervous system and stroke).

The comparative analysis of the Quemada model parameters between the control groups from the literature [30,33] and the studied patient group (Table 1) did not demonstrate statistically significant differences. This may be due to the relatively high discrepancy in the results in the study group and the presence of comorbidities affecting the final mean value. When analysing the values of the Quemada model parameters, a discrepancy in the results for all parameters was found. The value of the k_0_ parameter changed from the minimum value of 3.05 to the maximum one equal to 4.36, the k_∞_ parameter shifted from 1.48 to 1.90, and particularly high discrepancies were observed in the case of the γ′_c_ parameter changing from 1.53 to 15.16. In fact, the γ′_c_ parameter represents the moment of formation of red blood cell packets (rouleaux formation) [33,34,39,40]. The decreased whole-blood viscosity in the low shear rate range, i.e., in the range where aggregation is the dominant process [13,14,15], may imply the significance of this process in the studied patient group.

Despite many years of studies, the exact mechanisms of tinnitus generation are not fully explained. Therefore, in most cases the treatment of tinnitus is limited to reducing the symptoms. In the reviewed literature there are very few reports regarding rheological tests in patients suffering from this disorder. In the treatment of tinnitus and sudden hearing loss, drugs affecting the rheological properties of blood (rheologic infusion therapy) [39,41] are used to alleviate the microcirculatory disorders of the inner ear.

The present study analysed the results of blood rheological properties in patients complaining of tinnitus, as well as presenting other vascular, or metabolic diseases (Table 3). Whole blood viscosity, plasma viscosity, the tendency of erythrocytes to aggregate, and deformation depends not only on the type and duration of the disease, but also on comorbidities and administered medications [13,15,30,34,41].

Bearing in mind the abovementioned facts, the preliminary data are encouraging and indicate the occurrence of hemorheological changes in patients with tinnitus. Statistically significant differences were observed in the values of hemorheological parameters in the selected neurological diseases, both in relation to the control group and partially between patient groups. Therefore, the application of hemorheological tests in the diagnostic process of tinnitus may contribute to a better diagnosis and may be effective for further therapy [15,34].

## 5. Conclusions

The analysis of the results of hemorheological parameters in patients presenting with tinnitus indicated that:

- A reduced whole-blood viscosity in the low shear rate range is observed in the studied patient group as compared to the literature data;
- The results in the studied patient group may require additional subdivisions resulting from the presence of comorbidities.

The hemorheological tests may constitute an effective addition to the standard diagnosis of the tinnitus.

## Figures and Tables

**Table 1:** Mean values of hemorheological parameters for the study group of patients experiencing tinnitus. The variables k_∞_ and k_0_ in the Quemada formula (1) are interpreted as measures of erythrocyte stiffness and the degree of aggregation, whereas k_Q_ corresponds to the so-called erythrocyte intrinsic viscosity accounting for erythrocyte shape variation.

Rheological Parameters	Mean Value; *n* = 12
Haematocrit (%)	0.44 ± 0.01
Plasma viscosity η_p_ (mPas)	1.32 ± 0.04
Relative blood viscosity η_rel_ for γ′ = 0.1 s^−1^	20.0 ± 3.0
Relative blood viscosity η_rel_ for γ′ = 1 s^−1^	10.0 ± 0.8
Relative blood viscosity η_rel_ for γ′ = 10 s^−1^	4.9 ± 0.3
Relative blood viscosity η_rel_ for γ′ = 100 s^−1^	3.23 ± 0.01
k_0_	3.94 ± 0.11
k_∞_	1.71 ± 0.04
γ′_c_	6.0 ± 2

**Table 2:** Mean values of biochemical parameters in the study group LDL and HDL stand for low- and high-density lipoprotein, respectively.

Biochemical Parameters	Mean Value; *n* = 12
Glucose [mg/dl]	91,9 ± 1,5
Cholesterol [mg/dl]	188 ± 21
LDL [mg/dl]	116 ± 16
HDL [mg/dl]	53 ± 7
Triglycerides [mg/dl]	116 ± 12
Fibrinogen [mg/dl]	274 ± 16

**Table 3:** Characteristics of the selected audiological parameters and comorbidities in the study group. Average hearing threshold calculated from audiogram for frequency values of 0.5 kHz, 1 kHz, 2 kHz, 4 kHz, 6 kHz, and 8 kHz. Normal hearing thresholds: <20 dB at each of the frequencies tested.

No.	Avarage Hearing Threshold Right Ear [dBHL]	Average Hearing Threshold Left Ear [dBHL]	Tinnitus Location	Audiometric Results	Comorbidities
1.	20	20	right ear	normal	anxiety disorders, mixed headache, hypertriglyceridemia
2.	47	47	head	binaural hearing impairment	cochlear damage
3.	65	10	right ear	asymmetrical hearing impairment	Meniere’s disease
4.	40	40	head	bilateral high-frequency hearing impairment	post myocardial infarction, atherosclerotic lesions of the cephalic arteries, degeneration of the cervical spine, hypercholesterolemia
5.	70	60	left ear	binaural hearing impairment	anxiety disorders, hypercholesterolemia
6.	70	50	right ear	asymmetrical hearing impairment	polyneuropathy
7.	20	20	head	normal	hypercholesterolemia, hypertension, ischaemic brain lesions
8.	70	40	head	asymmetrical hearing impairment	ischaemic brain lesions, tortuous carotid arteries, hypercholesterolemia
9.	53	48	head	binaural hearing impairment	post-stroke, diabetes, hypertension, psoriasis, atherosclerotic lesions in the carotid arteries
10.	20	20	head	normal	multiple sclerosis
11.	20	20	right ear	normal	anxiety disorders, tension headache
12.	20	15	right ear	normal	anxiety disorders

## Data Availability

Data available on request.
